# Costo-efectividad de la oxigenación con membrana extracorpórea en pacientes con síndrome de dificultad respiratoria aguda en Colombia

**DOI:** 10.7705/biomedica.6386

**Published:** 2022-12-01

**Authors:** Álex García, Nelson Darío Giraldo

**Affiliations:** 1 Departamento de Anestesiología y Reanimación, Universidad de Antioquia, Medellín, Colombia Universidad de Antioquia Departamento de Anestesiología y Reanimación Universidad de Antioquia Medellín Colombia; 2 Departamento de Medicina Crítica y Cuidados Intensivos, Hospital Pablo Tobón Uribe, Medellín, Colombia Departamento de Medicina Crítica y Cuidados Intensivos Hospital Pablo Tobón Uribe Medellín Colombia; 3 Unidad de Cuidados Intensivos Cardiovascular, Clínica CardioVid, Medellín, Colombia Unidad de Cuidados Intensivos Cardiovascular Clínica CardioVid Medellín Colombia

**Keywords:** oxigenación con membrana extracorpórea, síndrome de dificultad respiratoria, respiración artificial, análisis costo-beneficio, Colombia, Extracorporeal membrane oxygenation, respiratory distress syndrome, respiration, artificial, cost-benefit analysis, mechanical ventilation, Colombia

## Abstract

**Introducción.:**

La terapia con oxigenación con membrana extracorpórea es costosa y, aunque existe existen indicios en la literatura de que puede ser una intervención costo-efectiva en los países desarrollados, hay dudas sobre su costo-efectividad en un país con un producto interno bruto per cápita bajo, como Colombia.

**Objetivo.:**

Determinar el incremento de la relación costo-efectividad de la terapia con oxigenación con membrana extracorpórea en pacientes con síndrome de dificultad respiratoria aguda en Colombia.

**Materiales y métodos.:**

Se eligieron pacientes adultos con diagnóstico de síndrome de dificultad respiratoria aguda para el análisis de costo-efectividad desde la perspectiva del sistema de salud. Se compararon aquellos pacientes con asistencia respiratoria mecánica con volúmenes bajos con aquellos tratados con oxigenación con membrana extracorpórea. Se determinaron los costos médicos directos de la atención y el incremento de la relación costo-efectividad a los 6 meses.

**Resultados.:**

El costo esperado por paciente en asistencia respiratoria mecánica protectora fue de COP$ 17’609.909. El costo del soporte mediante terapia de oxigenación con membrana extracorpórea fue de COP$ 98’784.116. La relación de costo-efectividad promedio fue de COP$ 141’662.435 por cada vida salvada (USD$ 41.276).

**Conclusiones.:**

El soporte con terapia de oxigenación con membrana extracorpórea tuvo un costo promedio de COP$ 141’662.435 por cada vida salvada, equivalente a USD$ 41.276 dólares y el incremento de la relación costo-efectividad fue de COP$ 608’783.750 (USD$ 177.384), casi diez veces superior a la regla de decisión de 3 PBI per cápita (COP$ 59’710.479).

## Abstract (en)

**Introduction:**

Extracorporeal membrane oxygenation therapy is expensive. There is evidence in the literature that it can be a cost-effective intervention in developed countries; however, in countries with low gross domestic product per capita, such as Colombia, there are still some doubts

**Objective:**

To determine the incremental cost-effectiveness ratio of extracorporeal membrane oxygenation in patients with acute respiratory distress syndrome in Colombia.

**Materials and methods:**

Cost-effectiveness analysis in healthcare in relation to adult patients diagnosed with acute respiratory distress syndrome with mechanical ventilation with low volumes compared to extracorporeal membrane oxygenation. The direct medical costs and the incremental cost-effectiveness ratio were determined at 6 months.

**Results:**

The expected cost per patient on protective mechanical ventilation was COP$ 17,609,909. The cost of extracorporeal membrane oxygenation therapy support in surviving patients was COP$ 98,784,116. The average cost-effectiveness ratio of extracorporeal membrane oxygenation was COP$ 141,662,435 for each life saved (USD$ 41,276).

**Conclusions:**

Support with extracorporeal membrane oxygenation therapy had an average cost of COP$ 141,662,435 for each life saved equivalent to USD$ 41,276. The incremental cost-effectiveness ratio COP$ was 608,783,750 (USD$ 177,384); almost ten times higher than the decision rule of three gross domestic product per capita (COP$ 59,710,479).

El síndrome de dificultad respiratoria aguda es un tipo de lesión pulmonar inflamatoria caracterizada por el daño del epitelio alveolar y endotelial, que incrementa la permeabilidad de la membrana alveolocapilar y produce edema pulmonar, lo que ocasiona hipoxemia grave e insuficiencia respiratoria aguda ^1^^,^^2^.

El síndrome de dificultad respiratoria aguda tiene una incidencia entre 7,2 y 86,2 casos por cada 100.000 pacientes-año, lo que corresponde al 10,4 % de los ingresos a la unidad de cuidados intensivos. Esto representa casi un cuarto (23,4 %) de los pacientes con asistencia respiratoria mecánica, con una mortalidad entre el 32 y el 61 % ^3^. En Colombia, Varón, *et al*., publicaron un estudio de cohorte con 70 pacientes en el que la edad media de presentación del síndrome de dificultad respiratoria aguda fue de 49 años y el 66 % eran hombres. La principal causa de este síndrome fue neumonía adquirida en la comunidad. La mortalidad a los 28 días fue del 34 % y los sobrevivientes tuvieron una mediana de 11 de días de asistencia respiratoria mecánica y una de 14 días de hospitalización en la unidad de cuidados intensivos ^4^.

El tratamiento de los pacientes con síndrome de dificultad respiratoria aguda consiste en corregir la causa subyacente y ofrecer soporte respiratorio con la estrategia de volúmenes bajos, acompañado de asistencia respiratoria en prono y relajación neuromuscular en los casos más graves ^5^^,^^6^. Para pacientes con hipoxemia persistente, la oxigenación con membrana extracorpórea es una técnica que permite oxigenar la sangre y eliminar el dióxido de carbono y, en casos adecuadamente seleccionados, ha demostrado aumentar la supervivencia de los pacientes con síndrome de dificultad respiratoria aguda grave ^7^^-^^10^.

En Colombia, hay al menos seis centros cardiovasculares con experiencia en la aplicación de este tratamiento ^11^. A pesar de esto, la oxigenación con membrana extracorpórea es una terapia costosa que ha demostrado ser una estrategia costo-efectiva en países con altos ingresos; sin embargo, hay dudas de su costo-efectividad en un país con un producto interno bruto (PIB) per cápita menor y con menos disponibilidad de recursos ^8^^,^^12^.

El objetivo de este estudio fue determinar, desde una perspectiva del sistema nacional de salud (tercer pagador), la relación costo-efectividad de la terapia de oxigenación con membrana extracorpórea en pacientes con síndrome de dificultad respiratoria aguda.

## Materiales y métodos

### Análisis de costo-efectividad desde la perspectiva del sistema nacional de salud

La población objetivo incluyó pacientes mayores de 18 años con diagnóstico de síndrome de dificultad respiratoria aguda, según los criterios de Berlín. Se excluyeron pacientes con edema pulmonar de origen cardiogénico, hipoxemia atribuida a hemorragia alveolar y enfermedad pulmonar crónica grave.

El horizonte temporal adoptado fue la duración del episodio agudo del síndrome de dificultad respiratoria aguda tratado en la unidad de cuidados intensivos y el total de la estancia hospitalaria. Este es un horizonte de tiempo adecuado para comparar las intervenciones, ya que el soporte respiratorio y la oxigenación con membrana extracorpórea solo se realizan en la unidad de cuidados intensivos. Como el horizonte de tiempo fue inferior a un año, no fue necesario utilizar tasa de descuento.

Las alternativas consideradas fueron el soporte de oxigenación con membrana extracorpórea en modalidad veno-venosa, en comparación con la asistencia respiratoria mecánica con volúmenes bajos asociada a la respiración en decúbito prono, con relajación neuromuscular o sin ella. Para elaborar el caso base, se siguieron los reportes de la literatura y las recomendaciones de las guías de práctica clínica y de un comité de expertos. Se asumieron 14 días de estancia en la unidad de cuidados intensivos para los pacientes con asistencia respiratoria mecánica sin diferencias entre los sobrevivientes y los fallecidos. Para los pacientes en oxigenación con membrana extracorpórea, se asumieron 14 días en perfusión, y una estancia en la unidad de cuidados intensivos de 24 días para los que sobrevivían y de 11 días para los que fallecían. Se asumió que los pacientes en oxigenación con membrana extracorpórea no presentaron complicaciones relacionadas con el tratamiento ^4^^,^^7^^,^^12^^-^^14^.

**Figura 1:**
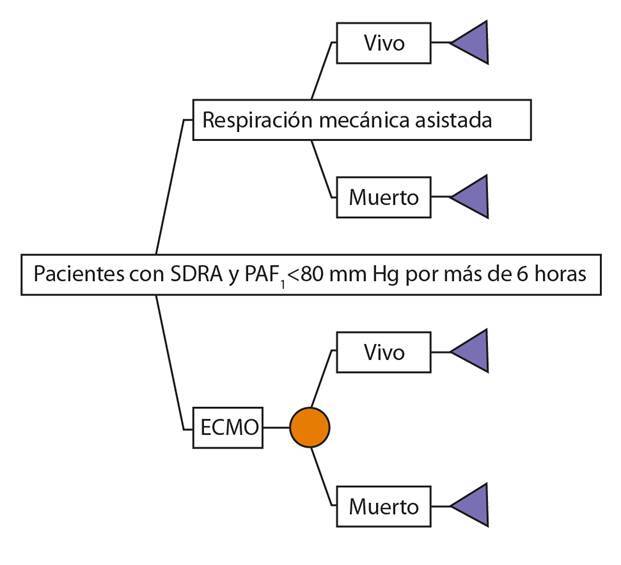
Árbol de decisiones

Para estimar los costos y beneficios esperados de ambas alternativas, se planteó un árbol de decisiones que refleja los diferentes resultados que puede experimentar un paciente (figura 1). En el árbol se asume que, una vez que el paciente tenga una PaO_2_/FiO_2_ menor de 80 mm Hg (con una presión arterial de CO_2_ mayor de 60 mm Hg por más de 6 horas o sin ella y después de recibir asistencia respiratoria mecánica con volúmenes bajos, PEEP adecuado y respiración en prono), puede continuar con asistencia respiratoria mecánica con volúmenes bajos o puede recibir soporte con oxigenación con membrana extracorpórea. Además, se asume que el paciente no tiene disfunción de otros órganos que requieran soporte, que lleva menos de 8 días con asistencia respiratoria mecánica invasiva y que el soporte con oxigenación con membrana extracorpórea se lleva a cabo en la misma institución por un grupo con experiencia en la terapia.

Como resultado de la efectividad, se consideraron los años de vida ganados a los 6 meses del evento. Las tasas de supervivencia se extrajeron de la revisión de Combes, *et al*., considerada de gran calidad metodológica, en la cual se reporta una supervivencia del 64 % con oxigenación con membrana extracorpórea comparada con el 52 % con asistencia respiratoria mecánica con volúmenes bajos ^9^.

Para establecer el listado de recursos costeados, se usó la metodología de caso tipo, elaborada con un especialista experto en el manejo de esta entidad clínica y en el soporte con oxigenación con membrana extracorpórea. Se hizo énfasis en los recursos utilizados en la atención directa de los pacientes, como estancia en la unidad de cuidado intensivo, estancia hospitalaria, estudios de laboratorio, estudios imagenológicos e insumos. Para la valoración monetaria, se utilizó el manual del SOAT (Seguro Obligatorio de Accidentes de Tránsito) del 2020 y el manual del ISS (Instituto de Seguros Sociales) del 2001 ajustado por el 30 %, de acuerdo con las recomendaciones del Instituto de Evaluación de Tecnologías en Salud (IETS) ^15^; posteriormente, se ajustó con el promedio del índice de precios al consumidor (IPC) después del 2012 hasta el 2020, con una media de 3,85. Para la valorización de los honorarios médicos y de la perfusión, se tomaron las tarifas de uno de los centros de una entidad aseguradora y se hizo un ajuste de costo a cuenta de 0,65, como lo recomienda Drummond ^16^. Las valoraciones monetarias de los insumos del soporte con oxigenación con membrana extracorpórea, se tomaron de los proveedores. Una vez hecho esto, se procedió a calcular los costos totales de la atención, como la suma de todos los costos implicados. No se analizaron los costos directos no médicos, como tampoco los costos indirectos y los costos futuros.

Como regla de decisión, se calculó el incremento de la relación costo-efectividad entre el soporte con oxigenación con membrana extracorpórea y la asistencia respiratoria mecánica, y se comparó esta relación con el valor correspondiente a tres PIB per cápita como umbral de aceptabilidad y disponibilidad de pagar, de acuerdo con las recomendaciones del IETS ^15^.

Para tener en cuenta la incertidumbre, se realizaron análisis de sensibilidad determinísticos y probabilísticos. En esto últimos, se modificó el costo de la oxigenación con membrana extracorpórea en un análisis de tipo umbral, en el cual el incremento de la relación costo-efectividad se comparó con un valor de tres PIB per cápita. Para la construcción del análisis de sensibilidad probabilístico, se asumieron distribuciones beta para las probabilidades con parámetro n y N, presentados en el cuadro 1. Para los costos directos, se utilizó una distribución triangular y se tomaron como mínimo los costos del manual del ISS del 2001 con los ajustes descritos y un máximo sobre la valoración de los procedimientos del manual SOAT del 2020.

**Cuadro 1:** Probabilidades de transición

Alternativa	Variable	Valor en el caso base	Parámetros de la distribución beta	Fuente, referencia
Asistencia respiratoria mecánica	Supervivencia	0,52	n=112; N=215	(9)
ECMO	Supervivencia	0,64	n=138; N=214	(9)

ECMO: *Extra Corporeal Membrane Oxigenation*

La construcción del árbol de decisión y los análisis estadísticos se llevaron a cabo con el programa Microsoft Excel 2016. El cambio de pesos colombianos (COP) a dólares (USD) se hizo mediante la equivalencia de COP$ 3.432 = USD$ 1, según la tasa representativa del mercado para el 31 de diciembre de 2020.

## Resultados

Los pacientes que recibieron soporte en la unidad de cuidados intensivos con oxigenación con membrana extracorpórea tuvieron costos mayores que aquellos tratados únicamente con asistencia respiratoria mecánica protectora. El costo esperado por paciente con esta asistencia respiratoria fue de COP$ 17’609.909, mientras que el costo del soporte con oxigenación con membrana extracorpórea en los pacientes que sobrevivieron fue de COP$ 98’784.116 y, para los que fallecieron, de COP$ 76’228.123. El costo total esperado del soporte con oxigenación extracorpórea fue de COP$ 90’663.959, lo cual resultó en un costo adicional de COP$ 73’054.050 (USD$ 21.286). De estos costos, el 34 % se debieron a los insumos utilizados en el soporte, el 23 % a la estancia en la unidad de cuidados intensivos y el 22 % a los honorarios por el soporte hemodinámico (cuadro 2).

**Cuadro 2:** Costos de las alternativas en pesos colombianos

**ISS 2001**	Asistencia respiratoria mecánica	*Extra corporeal Membrane oxigenation*	SOAT 2020	Asistencia respiratoria mecánica	*Extra corporeal membrane oxigenation*
Estancia	$ 16’146.946	$ 25’696.056	Estancia	$ 26’171.600	$ 40’716.600
Imagenológicos	$ 109.005	$ 1’621.204	Imagenológicos	$ 210.600	$ 3’436.500
Laboratorios	$ 1’168.773	$ 6’122.338	Laboratorios	$ 1’931.400	$ 10’519.600
Microbiológicos	$ 185.185	$ 185.185	Microbiológicos	$ 265.200	$ 265.200
Hemoderivados		$ 2’473.013	Hemoderivados		$ 4’986.200
Honorarios		$ 25’046.320	Honorarios		$ 25’046.320
Insumos		$ 37’640.000	Insumos		$ 37’640.000
Total	$ 17’609.909	$ 98’784.116	Total	$ 28’578.800	$ 122’610.420

La relación promedio de costo-efectividad de la oxigenación con membrana extracorpórea fue de COP$ 141’662.435 por cada vida salvada (USD$ 41.276) y el incremento de la relación costo-efectividad fue de COP$ 608’783.750 (USD$ 177.384), casi diez veces más alta que la regla de decisión de tres PIB per cápita (COP$ 59’710.479).

**Figura 2:**
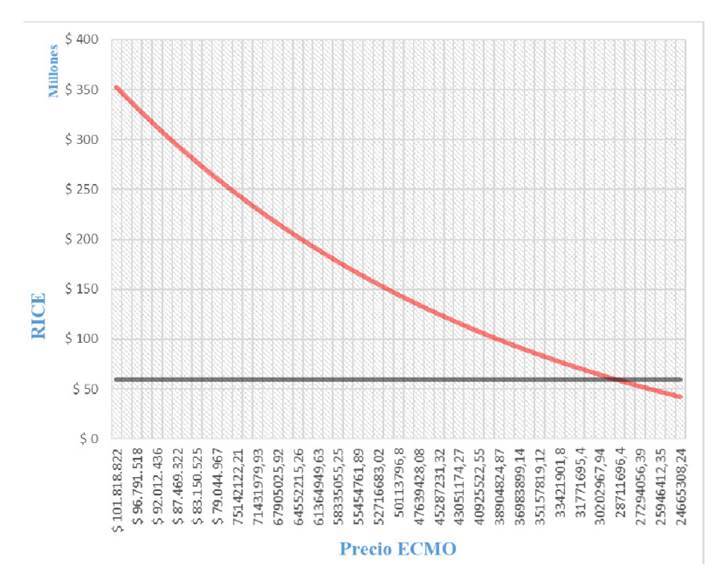
Análisis determinístico de umbral

Al hacer el análisis determinístico, los costos del soporte con oxigenación con membrana extracorpórea tendrían que ser de COP$ 30’824.741 para que los costos esperados fueran de COP$ 19’758.659 y el incremento de la relación costo-efectividad fuera de COP$ 58’897.262 por debajo de la regla de decisión (figuras 2 y 3).

**Figura 3:**
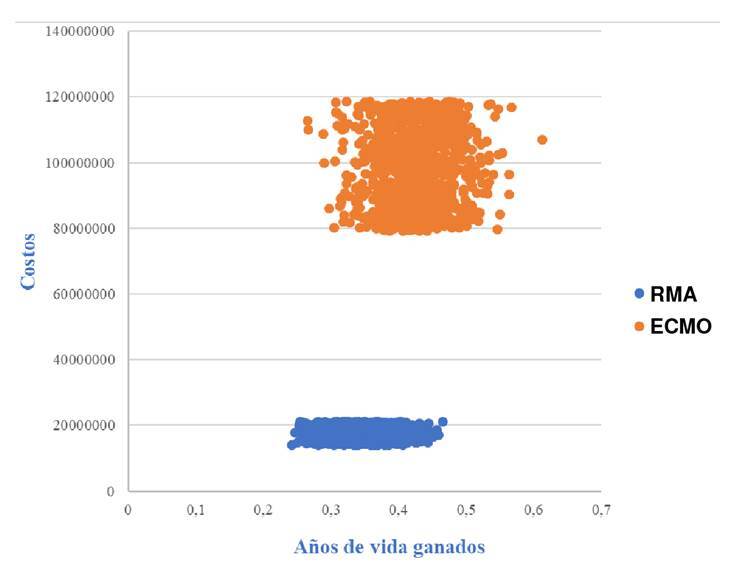
Gráfico de dispersión

**Figura 4:**
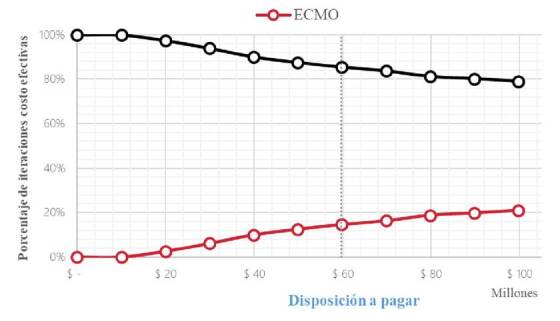
Curva de aceptabilidad

Al hacer el análisis de sensibilidad probabilístico, el soporte con oxigenación con membrana extracorpórea fue costo-efectivo en menos del 20 % de las simulaciones (figura 4).

## Discusión

Los resultados de este análisis económico sugieren que el soporte con oxigenación con membrana extracorpórea no es una estrategia dominante respecto al soporte con asistencia respiratoria mecánica protectora, en pacientes con síndrome de dificultad respiratoria aguda grave a los seis meses. Esta conclusión se mantiene en las diferentes circunstancias y análisis de sensibilidad, por lo que es muy probable que la incertidumbre en los parámetros y el modelo planteado no modifiquen el resultado.

Para el modelo se asumió un paciente base con síndrome de dificultad respiratoria aguda grave que no experimentaba complicaciones durante la estancia en la unidad de cuidados intensivos; sin embargo, estos pacientes pueden presentar complicaciones, como infecciones hospitalarias y disfunción de otros órganos que impactan el resultado clínico y los costos. Similarmente, los pacientes en oxigenación con membrana extracorpórea pueden tener complicaciones inherentes a esta terapia, como isquemia y sangrado en el sistema nervioso central, hemorragia gastrointestinal e infecciones hospitalarias, así como también, problemas técnicos como la falla de la membrana, y disfunción del circuito y de las cánulas, lo que se asocia con aumento de los costos del tratamiento.

Estos resultados coinciden con dos análisis de costo-efectividad previos, realizados en el Reino Unido y Canadá. El estudio CESAR, fue un estudio clínico llevado a cabo en Inglaterra y el Reino Unido en el 2009, en el cual se evaluaron la seguridad, la eficacia clínica y el costo-efectividad de la oxigenación con membrana extracorpórea comparado con el de la asistencia respiratoria mecánica protectora con volúmenes bajos. La evaluación económica adoptó la perspectiva del sistema nacional de salud y de la sociedad, con un horizonte de tiempo de seis meses. Se estimaron los costos directos, médicos y no médicos, de la atención mediante microcosteo. Se calcularon los años de vida ajustados a calidad (AVAC) para los sobrevivientes a los seis meses mediante el instrumento EuroQol-5D. En este estudio, se calcularon los AVAC ganados para toda la esperanza de vida de los sobrevivientes. La esperanza de vida se determinó con las tablas de vida específicas para la población del Reino Unido y Gales, y se asumió que los pacientes tenían una mejoría de su calidad de vida hasta los dos primeros años. Los costos promedio en salud fueron más altos en los pacientes asignados a oxigenación con membrana extracorpórea, con un incremento en el costo de GBP£ 40.544 (GBP£ 73.979 *versus* GBP£ 33.435) y el incremento de la relación costo-efectividad a los seis meses fue de GBP£ 250.162. A los seis meses, la diferencia de AVAC entre las dos alternativas fue de 0,03, pero, al hacer la proyección para la esperanza de vida, la diferencia en AVAC fue de 3,34 a favor del soporte con oxigenación extracorpórea con una relación de costo-utilidad de GBP£ 19.000 por AVAC, con más del 50 % de probabilidad de ser costo-efectivo con una disponibilidad a pagar de GBP£ 33.000 por AVAC ^12^.

Barret, *et al*., publicaron, en el 2018, un análisis de costo-beneficio de la oxigenación con membrana extracorpórea desde la perspectiva del sistema de salud canadiense. Se utilizó un modelo de transición de estados de una cohorte hipotética de pacientes. La edad al comienzo del modelo fue de 45 años, cada ciclo tenía una duración de un año y el modelo corrió hasta la muerte de los sujetos, con un horizonte de tiempo de toda la esperanza de vida. Los datos de costos para los pacientes con oxigenación con membrana extracorpórea y asistencia respiratoria mecánica durante el primer año, fueron tomados del CESAR ^12^, además del metaanálisis de Zampieri, *et al*. ^17^, y los costos después del año para los pacientes se tomaron del estudio publicado por Herridge, *et al*. ^18^.

Para el cálculo de los AVAC para los sobrevivientes de síndrome de dificultad respiratoria aguda, se tomó como utilidad aquella publicada por Skinner, *et al*. ^19^; en ese estudio, se asumió un aumento de la calidad de vida hasta el quinto año después del egreso, luego del cual, la calidad de vida se igualaba a la de la población general. La oxigenación con membrana extracorpórea se asoció con una ganancia de 5,2 años de vida adicionales y 4,05 AVAC, en comparación con la asistencia respiratoria protectora; el incremento del costo fue de CAD$ 145.967 y un el incremento de la relación costo-efectividad de CAD$ 36.000 CAD/AVAC, lo cual fue costo-efectivo con una disponibilidad a pagar de 50.000 CAD/AVAC ^8^.

Durante la pandemia del COVID-19, hubo gran demanda de centros de oxigenación con membrana extracorpórea en el mundo. La tasa de supervivencia global en los metaanálisis sobre dicha condición, fue del 63 %, similar a la de las otras causas del síndrome de dificultad respiratoria aguda grave en oxigenación extracorpórea, lo que sugiere que estos resultados también podrían aplicarse a este grupo de pacientes ^20^.

En el presente análisis económico, solo se tomaron en cuenta los costos directos médicos de la atención en la unidad de cuidados intensivos y hospitalaria. Esto se debe a que el objetivo del análisis era conocer el incremento en el costo de la terapia por vida salvada y el incremento de la relación costo-efectividad, con el propósito de generar un dato concreto para la toma de decisiones para el tercer pagador. Es claro que, aunque los pacientes y familiares incurren en costos que pueden ser diferentes para las intervenciones, estos, al hacer parte de los gastos de bolsillo, no influencian la decisión del tercer pagador.

De forma similar, los sobrevivientes del síndrome de dificultad respiratoria aguda incurren en costos médicos futuros, como rehabilitación y consultas de revisión que no fueron tomados en cuenta en el análisis, ya que no son diferentes para las intervenciones y, de esta forma, no influencian la toma de decisiones.

Un punto controvertido del análisis es el tiempo de valoración de los resultados de efectividad. En el presente análisis, se tomó un horizonte de tiempo de seis meses. Los pacientes que recibieron soporte con oxigenación con membrana extracorpórea tuvieron una mayor supervivencia en este punto, en comparación con los pacientes que continuaron con asistencia respiratoria mecánica; esta diferencia, aunque con significancia estadística, fue pequeña en total de años de vida ganados.

Por esta razón, la estimación del incremento de la relación costo-efectividad fue elevada y por encima de la regla de decisión de tres veces el PIB per cápita. Este hallazgo fue idéntico al del estudio CESAR, en el cual el incremento de la relación costo-efectividad a los seis meses estuvo muy por encima de la disponibilidad para pagar; sin embargo, los estudios publicados hasta la actualidad muestran que los pacientes que sobreviven al síndrome de dificultad respiratoria aguda mantienen este beneficio en supervivencia en el tiempo y presentan una mejoría de su calidad de vida en los primeros cinco años después del egreso del hospital, cuando se equipara la calidad de vida con la de la población general. En el estudio CESAR y en el de Barrett, *et al*., modelaron esta diferencia de efectividad para toda la esperanza de vida de los pacientes y encontraron una diferencia importante en los años de vida ganados y en los AVAC, entre las intervenciones. Al utilizar estos beneficios en el cálculo del incremento de la relación costo-efectividad, la oxigenación con membrana extracorpórea estuvo dentro de la disponibilidad de pagar y fue recomendada como una estrategia costo-efectiva.

Con esta consideración y a modo de ejercicio, se hizo un modelado de Markov simulando las diferencias entre la supervivencia de la oxigenación con membrana extracorpórea y la asistencia respiratoria mecánica en un periodo de cinco años, para un paciente de 40 años. Se utilizaron las curvas de supervivencia y las tablas de vida para la población colombiana publicadas por el DANE para el año 2020. Los resultados de este modelo exploratorio mostraron que los pacientes con asistencia respiratoria mecánica tuvieron una ganancia de 6,5 años y, aquellos con oxigenación extracorpórea, de 7,9 años, con una diferencia de 1,4 años entre las dos intervenciones. Aplicando esta diferencia de efectividad al incremento del costo, el incremento de la relación costo-efectividad sería de COP$ 52’181.464, que se ubica por debajo de la regla de decisión. Sin embargo, este hallazgo debe interpretarse con precaución, ya que no se tuvieron en cuenta los costos de un soporte prolongado con oxigenación extracorpórea, ni el costo de las complicaciones posibles, así como tampoco, los costos futuros de los sobrevivientes.

La principal limitación del estudio es no contar con datos locales sobre la efectividad del soporte con oxigenación con membrana extracorpórea para el cálculo del incremento de la relación costo-efectividad. En el análisis, se utilizaron los datos publicados en el metaanálisis publicado por Combes, *et al*., que es la mejor información disponible hasta el momento ^9^. En la Clínica Cardio Vid, el principal centro de soporte de oxigenación con membrana extracorpórea en Medellín, desde el año 2013 hasta el año 2021, más de 250 pacientes han recibido este tratamiento, con una supervivencia de alrededor del 60 %, dato que debe evaluarse con precaución dado que se incluyen pacientes con choque cardiogénico y soporte con oxigenación extracorpórea venoarterial que tienen una supervivencia diferente. Estas tasas de supervivencia están por debajo de las reportadas por Combes, lo que modificaría los resultados del incremento de la relación costo-efectividad (datos proporcionados por la Clínica Cardio VID, Medellín).

Los análisis de sensibilidad del incremento de la relación costo-efectividad fueron muy influenciados por la efectividad de la oxigenación con membrana extracorpórea. Los tomadores de decisiones deben tener esta información en cuenta ya que en centros donde la supervivencia con oxigenación extracorpórea sea inferior al 64 %, el posible costo-efectividad de esta terapia puede estar comprometido.

En conclusión, el soporte con oxigenación con membrana extracorpórea en pacientes con síndrome de dificultad respiratoria aguda grave, es más costoso que la asistencia respiratoria mecánica protectora, con un costo de COP$ 141’662.435 (USD$ 41.276) por cada vida salvada y a los seis meses; su relación de costo-efectividad está muy por encima de la regla de decisión de tres PIB per cápita.
